# Examining the joint impact of missing data mechanisms and item parameter drift on the accuracy of item response theory-based test equating

**DOI:** 10.1371/journal.pone.0353665

**Published:** 2026-07-29

**Authors:** Ayse Bilicioglu Gunes, Serife Zeybekoglu Yesil

**Affiliations:** 1 Department of Educational Measurement and Evaluation, Faculty of Education, Bartin University, Türkiye; 2 Department of Educational Measurement and Evaluation, Ministry of National Education, Türkiye; Takushoku University, JAPAN

## Abstract

Maintaining score comparability across different test administrations is essential in large-scale educational and psychological assessment. Two major threats to equating accuracy are item parameter drift (IPD), reflecting changes in anchor item characteristics over time, and missing item responses, which commonly occur in operational testing. Although each factor has been studied separately, their joint impact on equating accuracy has not been systematically evaluated. A Monte Carlo simulation was conducted using a nonequivalent groups with anchor test design. Data were generated under a three-parameter logistic model with 2,000 examinees per form and 500 replications per condition. The Stocking–Lord method was used to estimate equating constants across 25 conditions, varying IPD rate (0%, 10%, 20%), IPD magnitude (0, 0.25, 0.50), missing data mechanism (MCAR vs. MAR), and missing data rate (0%, 10%, 20%). Missing responses were addressed using multiple imputation by chained equations. Equating accuracy was evaluated using bias and root mean square error. Results indicated that the *B* constant was highly sensitive to the missing data mechanism: MCAR conditions produced near-zero bias, whereas MAR conditions introduced substantial positive bias, particularly at higher missing rates. IPD alone did not result in meaningful bias but increased estimation variability. When IPD and MAR co-occurred, equating error exceeded the sum of their individual effects, indicating an interaction rather than a purely additive relationship. Although multiple imputation reduced error under MCAR, it did not fully eliminate bias under MAR conditions. In contrast, the scale constant remained stable across all conditions. Overall, the missing data mechanism had a stronger impact on equating accuracy than either the rate of missingness or the degree of item drift alone. When both factors were present, their combined influence led to increased error that could not be fully corrected through imputation. These findings underscore the importance of carefully evaluating missing data mechanisms, selecting appropriate imputation strategies, such as MICE, and monitoring anchor item stability to ensure accurate score equating, particularly in psychological assessment contexts where even small errors may affect individual-level decisions.

## Introduction

In educational and psychological measurement, the use of multiple test forms that assess the same construct is a common practice, particularly to prevent item exposure, ensure test security, and administer large-scale tests. When different forms are administered to different groups of examinees, it is necessary to place the resulting scores on a common scale to enable meaningful comparisons. In psychological assessment, this issue of comparability goes beyond a simple technical concern. When the same construct is measured across different settings or groups, a lack of score consistency can seriously weaken the validity of any clinical or diagnostic interpretations based on those results. This process is referred to in the literature as test equating [1] and can also be understood as expressing examinees’ ability parameters on the same latent trait metric [2]. Through equating, it is possible to monitor individuals’ performance across administrations and to compare item parameter estimates obtained from different samples. In psychological assessment contexts, such comparability is essential, as test scores are often used to inform decisions about individuals’ cognitive, emotional, or behavioral functioning.

Item Response Theory (IRT) based equating approaches are widely used in measurement practice because they model item and person parameters separately and are relatively less sensitive to sampling fluctuations [3–5]. This is particularly important in psychological measurement, where ensuring comparability across administrations supports valid inferences about latent traits. One of the most employed designs in this context is the nonequivalent groups with anchor test (NEAT) design [1], in which a set of common items is used to link a new form to a base form and place both on the same scale [6]. This approach is supported by evidence showing that characteristic-curve methods based on separate calibration yield more stable results than moment methods under the Common-Item Equating Design [7].

In IRT-based equating, anchor items play a central role in establishing the linking, and the accuracy of the linking constants largely depends on the stability of their item parameters. Accordingly, the validity of the equating results rests on the assumption that anchor items function equivalently across forms. Item parameter drift (IPD) refers to the phenomenon in which the difficulty or discrimination parameters of anchor items shift across administrations [8,9], thereby posing one of the most fundamental threats to the assumption of parameter invariance. The primary causes of IPD include curriculum changes, item exposure, and cultural, educational, or technological developments that may render anchor items easier or harder for examinees over time [10,11]. When item parameter drift occurs, scale transformations may become biased, which can in turn affect ability estimates and compromise the comparability of scores across test forms [12]. Among the various forms of IPD, drift in the difficulty (b) parameter represents the most encountered and practically consequential form in large-scale assessments [13,14]. Although drift in the discrimination (a) and pseudo-guessing (c) parameters also occurs in practice, b-parameter drift appears to be more prevalent in operational testing contexts and has been shown to have a more direct impact on equating accuracy. Drift in a parameter tends to be less frequent and has comparatively more localized effects on scale transformation, while c-parameter drift is particularly difficult to detect reliably due to the inherently lower precision of its estimates [13,14]. For these reasons, the present study focuses specifically on b-parameter drift, reflecting conditions most likely to arise in operational testing contexts. In psychological testing contexts, such distortions may compromise the accuracy of individual-level interpretations, especially when test scores are used for diagnostic or evaluative purposes.

Another major challenge in equating studies is missing data. Missing responses are ubiquitous in social and educational research and directly affect the validity and reliability of statistical analyses. Examinees may omit items for various reasons, such as difficulty in choosing among response options, lack of motivation to respond, concerns about data security, or time limitations that prevent them from reaching all items [15]. The impact of missing data is determined not only by the proportion of missingness but also by its pattern and underlying mechanism [16]. As the amount of missing data increases, the representativeness of the sample decreases, generalizability is compromised, and statistical bias tends to increase [17]. In psychological research and assessment, such biases may disproportionately affect certain groups of individuals, thereby raising concerns about fairness and validity. Following Rubin [18], missing data mechanisms are categorized as missing completely at random (MCAR), missing at random (MAR), and missing not at random (MNAR). Under MCAR, missingness is independent of both observed and unobserved variables; under MAR, it depends only on observed variables such as ability level [19]. In this study, only MCAR and MAR were simulated, since MNAR introduces complex methodological challenges in large-scale test equating [20,21]. MCAR represents completely random missingness, whereas MAR reflects missingness dependent on observed variables, consistent with patterns observed in empirical data [15].

To address missing data, a variety of traditional and modern approaches have been proposed. Traditional methods such as listwise and pairwise deletion may lead to biased estimates and inflated standard errors, particularly when the proportion of missing data is high [22]. Among modern techniques, multiple imputation (MI) is a flexible and efficient approach that involves generating multiple plausible versions of the incomplete dataset and combining results from these completed datasets. Allison [23] noted that MI is applicable across a wide range of statistical models, making it one of the most practical imputation methods. In recent years, MI has been increasingly recommended in the IRT context to reduce bias due to missing data and to provide more realistic representations of estimation uncertainty [24]. In the present study, MICE was applied to generate multiple plausible datasets and to combine estimates using Rubin’s rules [23,25]. The number of imputations for each condition is reported in the Method section, where the rationale for the selected imputation strategy is described in detail.

A review of the literature indicates that, although test equating has been examined in relation to missing data [26–28] and item parameter drift [29–33] separately, to our knowledge, no study has yet examined their interactive effects within a single controlled framework, a gap that has direct implications for operational equating practice.

Given that missing responses are unavoidable in operational testing programs, understanding how missing data and anchor item parameter drift jointly affect equating accuracy is of both theoretical and practical importance. In particular, it remains unclear how different missing data mechanisms and rates influence the standard errors of the equating constants in the presence of parameter drift, to what extent MI can reduce this uncertainty, and how the performance of the commonly used equating method is affected under these non-ideal conditions.

The current simulation study was designed to systematically manipulate missing data mechanism (MCAR, MAR), missing data rate (0%, 10%, 20%), IPD rate (0%, 10%, 20%), and IPD magnitude (0, 0.25, 0.50), allowing for a controlled examination of equating accuracy under realistic operational scenarios using the Stocking–Lord (SL) method and IRT true score equating. The SL method was selected over competing linking methods, notably the Mean/Sigma and Haebara approaches, due to its demonstrated robustness in the presence of IPD. Unlike the Mean/Sigma method, which relies on the mean and standard deviation of item parameter estimates and may therefore be more sensitive to parameter drift, the SL method minimizes discrepancies across the full test characteristic curve, thereby reducing sensitivity to localized parameter instability. The Haebara method similarly operates at the item level but has, in some simulation studies, shown sensitivity to outlying item parameters [29]. Previous studies have also suggested that the SL method can exhibit relatively greater stability under conditions such as small sample sizes and noisy data [34,35]. In combination with IRT true score equating, the SL method facilitates the transformation of item parameters and ability estimates across test forms. This is particularly important when the 3PL model is used, as non-zero guessing parameters (c) require accurate scale alignment.

In sum, the study integrates NEAT design, multiple imputation for missing data, and the SL scaling approach to investigate the joint effects of IPD and missing responses on equating constants (A and B), where A represents the scale transformation constant, and B represents the location transformation constant used to place the new form on the base-form metric [1].

To the authors’ knowledge, research examining the joint effects of item parameter drift and missing data within a unified simulation framework in IRT-based equating remains limited. The findings are intended to provide evidence-based guidance to practitioners and researchers who must make methodological decisions regarding anchor item management and missing data handling in operational testing programs.

### Purpose of the study

The present study aims to examine the joint effects of item parameter drift and missing data on the accuracy of IRT true score equating within a unified simulation framework. Specifically, the study investigates how varying levels of anchor item drift (differing in both proportion and magnitude) interact with different missing data mechanisms and rates to influence the bias and precision of SL equating constants. A secondary aim is to evaluate the extent to which multiple imputation mitigates equating error under these non-ideal conditions. By systematically manipulating these factors, the study seeks to provide evidence-based guidance for methodological decisions in operational equating practice. Beyond its methodological contribution, the study aims to inform best practices in psychological assessment by identifying conditions under which equating procedures may produce biased or unstable results.

Based on this purpose, the study addresses the following research questions:

1. How does anchor item parameter drift, in terms of both the proportion of drifting items and drift magnitude, affect the bias and Root Mean Square Error (RMSE) of SL equating constants?
2. How do the missing data mechanisms (MCAR vs. MAR) and the missing data rate influence the bias and RMSE of equating constants?
3. What are the joint effects of item parameter drift and missing data on equating accuracy, and do these combined effects exceed those observed when each source of error is present in isolation?
4. To what extent does MI reduce bias and improve the precision of equating constants under missing data conditions, and does this effect vary in the presence of item parameter drift?

## Method

### Research design

This study employed a simulation-based research design as described by Dooley [36]. Unlike empirical studies, which are constrained by observed data, simulation approaches allow researchers to systematically manipulate the parameters and conditions of interest and examine their behavior under controlled scenarios. In this context, a Monte Carlo simulation framework was used to investigate the effects of common item parameter drift and missing data conditions on IRT-based test equating results.

**Ethics statement.** Ethics approval was not required because this study used simulated data and did not involve human participants.

### Data generation

Data sets were generated in R (version 4.5.1) to evaluate the performance of the test equating method under varying missing-data mechanisms and IPD conditions. A Monte Carlo simulation approach was adopted, with 500 replications following common practice in the simulation literature [37]. A sample of 2,000 examinees was simulated for each form in each replication. The data generation code was reviewed and test-run prior to the main simulation to confirm that the generated parameters were consistent with the specified distributions. Data generation was based on the three-parameter logistic (3PL) model within the IRT framework.

### Item parameters

Item difficulty parameters (b) were generated from a uniform distribution U(−2, 2), a range commonly used in simulation studies to represent tests of moderate difficulty [38]. Item discrimination parameters (a) were generated from a log-normal distribution, log(a) ~ N(0, 0.5), where 0.5 denotes the standard deviation of the log-normal distribution, with the additional constraint that no item had a discrimination value below 0.4 [5]. The use of a log-normal distribution ensures that discrimination parameters remain positive and reflect the asymmetric distributions typically observed in real test items [38].

The guessing parameter (c) was fixed at 0.20 for all items, consistent with chance performance on five-option multiple-choice items. In the 3PL model, the pseudo-guessing parameter is often treated as a fixed item-level constant, and fixing it is common due to instability in its estimation [39]. It should be acknowledged that fixing c at a single value across all items is a simplifying assumption; in practice, pseudo-guessing parameters vary across items and are rarely known with certainty. Additional design assumptions, including the use of a fixed pseudo-guessing parameter and unidirectional drift, may limit the generalizability of the findings to more complex operational testing conditions.

### Ability distribution

Examinee ability parameters (θ) were generated from a standard normal distribution, N(0, 1) [40]. Sass et al. [41] noted that a normally distributed ability parameter is commonly assumed for stable estimation in IRT models. This assumption is also consistent with many previous simulation studies [14,30]. Ability parameters were estimated using the Expected a Posteriori (EAP) method, which has been shown to provide more accurate estimates compared to maximum likelihood estimation in unidimensional IRT frameworks [41,42].

### Test design

The test length for both the base and new forms was set to 40 items, consistent with conditions commonly used in IRT simulation studies [14,39]. This length also reflects the typical number of items found in large-scale standardized assessments, such as certification and licensure examinations. An additional reason for selecting this length is that it conveniently meets the minimum anchor-item proportion required in a NEAT design.

Each form included 8 internal anchor items (20%). Kolen and Brennan [1] recommend that, in a NEAT design with 40 items, at least 20% of the test items be anchor items.

Within the scope of the study, the proportion of drifting anchor items was set at 10% and 20%. Accordingly, when the data were manipulated, drift was introduced to the 1 and 2 anchor items, respectively. These numbers are consistent with the number of drifting items observed in operational testing settings [13].

### Experimental conditions

#### IPD conditions.

IPD conditions were defined based on factors expected to influence equating error. Three main dimensions were considered: (1) the proportion of drifting items, (2) the type, direction, and magnitude of drift, and (3) the parameter in which the drift occurs.

In this study, the proportion of drifting items in the anchor set was manipulated at three levels: 0% (control), 10%, and 20%. This range is consistent with both simulation studies and empirical findings on the number of misbehaving common items in operational testing settings [13,14].

IPD was applied only to the difficulty (b) parameter, consistent with previous simulation studies that focused on b-parameter drift in isolation [30,43]. This approach allows for a cleaner examination of the effect of difficulty drift on equating results.

IPD was simulated as unidirectional (positive), with all drifting anchor items having their b values increased in the same direction. Wells et al. [14] stated that unidirectional drift has a greater impact on ability estimation compared to bidirectional drift. In bidirectional cases, positive and negative deviations may partially cancel each other out, potentially masking the true effect of IPD. The decision to apply drift exclusively in the positive direction was made to facilitate the detectability of equating error and to isolate the effect of drift magnitude. Accordingly, the findings may be most applicable to testing conditions in which drift occurs predominantly in a single direction.

The magnitude of IPD was set at b = 0.0, 0.25, and 0.50, representing no drift, small drift, and moderate drift, respectively [14,30].

### Missing data conditions

In this study, MCAR and MAR mechanisms were simulated as missing data conditions. Unlike MNAR, in which missing observations follow a systematic pattern associated with the latent levels of the measured variable, MCAR and MAR are considered more tractable mechanisms that allow for valid inference when appropriately handled [19,44]. MNAR was excluded due to the methodological complexity associated with modeling and interpreting it in large-scale test equating contexts [20,21].

Under the MAR condition, examinees were first divided into three groups based on their latent ability (θ) (low, medium, and high) using tertile splits. The probability of missing responses was then assigned differentially across these groups. For the 10% missing rate condition, the probabilities were set at 0.15, 0.10, and 0.05 for the low, medium, and high ability groups, respectively. For the 20% missing rate condition, the corresponding probabilities were 0.30, 0.20, and 0.10. Although MAR is formally defined with respect to observed variables, latent ability (θ) was used in this simulation as a proxy for observed performance indicators, such as total score or prior achievement, which are typically highly correlated with ability in operational testing contexts [15,21]. This approach was intended to approximate realistic omission behavior, in which lower-performing examinees are more likely to omit items. Accordingly, the present implementation is best interpreted as a near-MAR condition rather than a strictly observed-variable MAR process.

The missing data rate was set at 0%, 10%, and 20%. These rates are consistent with typical missing data patterns observed in large-scale assessments such as Programme for International Student Assessment (PISA), where missing item response rates have been reported to average approximately 8% and reach up to 19% across countries [15,45], and were further supported by simulation studies examining similar conditions in IRT and equating contexts [21,26,46].

### Simulation design structure

The simulation design in this study included the factors of IPD rate (0%, 10%, 20%), IPD magnitude (0, 0.25, 0.50), missing data mechanism (MCAR, MAR), and missing data rate (0%, 10%, 20%).

However, the design was not fully crossed. Certain combinations were excluded based on logical constraints. For example, when the missing data rate was 0%, the missing data mechanism (MCAR/MAR) was not applicable. Similarly, when the IPD rate was 0%, the IPD magnitude factor was not implemented. Therefore, instead of generating all possible combinations, only theoretically meaningful and interpretable conditions were included. In total, 25 experimental data sets were created. Detailed information on these conditions is provided in Table 1.

**Table 1:** Simulation conditions (N = 25 conditions × 500 replications).

Condition	IPD Rate	IPD Magnitude (Δb)	Missing Data Mechanism	Missing Data Rate	Description
	(0% / 10% / 20%)	(0.00 / 0.25 / 0.50)	(MCAR / MAR)	(0% / 10% / 20%)	
**K1**	**0%**	**0.00**	**---**	**0%**	**Full control (no IPD, no missing data)**
K2	10%	0.25	---	0%	IPD only (low rate, small magnitude)
K3	10%	0.50	---	0%	IPD only (low rate, moderate magnitude)
K4	20%	0.25	---	0%	IPD only (moderate rate, small magnitude)
K5	20%	0.50	---	0%	IPD only (moderate rate, moderate magnitude)
K6	0%	0.00	MCAR	10%	Missing data only — MCAR (low rate)
K7	0%	0.00	MCAR	20%	Missing data only — MCAR (moderate rate)
K8	0%	0.00	MAR	10%	Missing data only — MAR (low rate)
K9	0%	0.00	MAR	20%	Missing data only — MAR (moderate rate)
K10	10%	0.25	MCAR	10%	IPD (low/small) × MCAR (low)
K11	10%	0.25	MCAR	20%	IPD (low/small) × MCAR (moderate)
K12	10%	0.50	MCAR	10%	IPD (low/moderate) × MCAR (low)
K13	10%	0.50	MCAR	20%	IPD (low/moderate) × MCAR (moderate)
K14	10%	0.25	MAR	10%	IPD (low/small) × MAR (low)
K15	10%	0.25	MAR	20%	IPD (low/small) × MAR (moderate)
K16	10%	0.50	MAR	10%	IPD (low/moderate) × MAR (low)
K17	10%	0.50	MAR	20%	IPD (low/moderate) × MAR (moderate)
K18	20%	0.25	MCAR	10%	IPD (moderate/small) × MCAR (low)
K19	20%	0.25	MCAR	20%	IPD (moderate/small) × MCAR (moderate)
K20	20%	0.50	MCAR	10%	IPD (moderate/moderate) × MCAR (low)
K21	20%	0.50	MCAR	20%	IPD (moderate/moderate) × MCAR (moderate)
K22	20%	0.25	MAR	10%	IPD (moderate/small) × MAR (low)
K23	20%	0.25	MAR	20%	IPD (moderate/small) × MAR (moderate)
K24	20%	0.50	MAR	10%	IPD (moderate/moderate) × MAR (low)
K25	20%	0.50	MAR	20%	IPD (moderate/moderate) × MAR (moderate)
**Total**	**25 conditions**	**× 500 replications**	**= 12,500 simulations**		

***Note.*** *IPD = Item Parameter Drift; Δb = magnitude of drift in the b parameter; MCAR = Missing Completely at Random; MAR = Missing at Random.*

***Note.*** *When the IPD rate is 0%, IPD magnitude is not applicable (---). When the missing data rate is 0%, the missing data mechanism is not defined (---).*

### Data analysis

#### Parameter estimation.

Item parameters for both the base and new forms were estimated using the mirt package in R [47]. A unidimensional 3PL model was specified in all conditions. To ensure consistency across conditions and avoid estimation instability, the guessing parameter (c) was fixed at 0.20 in all analyses.

Examinee ability parameters (θ) were estimated using the Expected A Posteriori (EAP) method. EAP is one of the most widely used methods for ability estimation in unidimensional IRT [42] and has been shown to yield more accurate estimates than maximum likelihood (ML) methods [41].

### Missing data handling

Missing data were handled using Multiple Imputation by Chained Equations (MICE, [25]), implemented via the mice package in R [48]. Given the binary (0/1) nature of item response data, logistic regression was used as the imputation model for each item within the MICE framework. This choice is consistent with recommendations for imputing dichotomous variables [25].

The number of imputations (M) was set equal to the percentage of missing data (e.g., M = 10 for 10% missingness). This approach is consistent with the rule M ≥ percentage of missing data suggested by Graham et al. [49] and White et al. [50]. Following multiple imputation, item parameters were estimated separately for each imputed dataset using the mirt package.

### Equating procedure

Equating was conducted using the SL method within a NEAT design. Analyses were performed using the equateIRT package [51]. Within the SL framework, the equating constants A and B are estimated by minimizing the weighted sum of squared differences between the test characteristic curves of the base and new forms. Item parameters from the new form are then transformed onto the base-form metric. Under the 3PL model, this transformation rescales the a and b parameters while maintaining consistency in the interpretation of the c parameter across forms [1,52].

In each replication, the new form was equated to the base form. Common items (Q1–Q8) were automatically identified using the item name matching feature of the equateIRT package. In the presence of missing data, pooled parameter estimates were used.

### Evaluation metrics

The estimation of the A and B transformation constants obtained from the SL method was evaluated using Bias and RMSE. These metrics are widely used as standard performance indicators in IRT equating simulation studies [53]. Bias and RMSE were calculated as follows:


$$\begin{array}{c} \text{Bias}=\frac{\text{1}}{R}\sum_{r=\text{1}}^{R}({\hat{\lambda }}_{r}-\lambda ) \end{array}$$

$$\begin{array}{c} \text{RMSE}=\sqrt{\frac{\text{1}}{R}\sum_{r=\text{1}}^{R}({\hat{\lambda }}_{r}-\lambda {)}^{\text{2}}} \end{array}$$

where λ̂_r is the estimated parameter in replication r, λ is the true parameter value, and R is the total number of replications.

In addition to absolute Bias and RMSE, Relative Bias (RB) and Relative RMSE (RRMSE) were also computed to assess the magnitude of deviation relative to the true parameter values [37,54]:


$$\begin{array}{c} \text{RB}=\frac{\text{Bias}}{\lambda }\times \text{1}\text{0}\text{0} \end{array}$$

$$\begin{array}{c} \text{RRMSE}=\frac{\text{RMSE}}{|\lambda |}\times \text{1}\text{0}\text{0} \end{array}$$

In calculating relative metrics, conditions with |λ| < 0.05 were excluded to avoid division-by-zero problems; for these cases, only absolute Bias and RMSE were reported. The threshold of |λ| < 0.05 was adopted as a practical cutoff to prevent numerically unstable relative metrics when the true parameter value approaches zero; this decision is consistent with common practice in equating simulation studies [54]. The criterion |RB| > 5%, proposed by Hoogland and Boomsma [55], was used as a threshold for identifying practically significant bias.

## Results

This section presents the bias and RMSE values for the A and B equating constants obtained using the SL method across four research questions. The values for all conditions are reported in Table 2, and the differences relative to the reference condition (K1) are presented in Table 3. Relative bias (RB_B) is reported in Table 2 only for conditions in which the true B constant meets the |B_true| ≥ 0.05 threshold; conditions falling below this threshold are marked with a dash (—) to avoid numerically unreliable results, and for these conditions only absolute bias and RMSE are interpreted.

**Table 2:** Bias, RMSE, and relative bias of the SL equating constants across 25 simulation conditions.

Condition	IPD Rate	Δb	Missing Mechanism	Missing Rate	Bias*A*	RMSE*A*	Bias*B*	RMSE*B*	RB*B* (%)
**K1 (Reference)**	0%	0.00	—	0%	0.0013	0.0512	−0.0007	0.0493	—
K2	10%	0.25	—	0%	0.0009	0.0535	−0.0011	0.0496	—
K3	10%	0.50	—	0%	−0.0014	0.0567	0.0020	0.0525	−3.14
K4	20%	0.25	—	0%	0.0002	0.0553	0.0014	0.0530	−2.25
K5	20%	0.50	—	0%	−0.0043	0.0634	0.0060	0.0580	−4.78
K6	0%	0.00	MCAR	10%	−0.0066	0.0523	0.0027	0.0490	—
K7	0%	0.00	MCAR	20%	−0.0125	0.0550	0.0039	0.0491	—
K8	0%	0.00	MAR	10%	0.0018	0.0522	0.0094	0.0499	—
K9	0%	0.00	MAR	20%	−0.0045	0.0535	0.0251	0.0566	—
K10	10%	0.25	MCAR	10%	−0.0078	0.0547	0.0028	0.0498	—
K11	10%	0.25	MCAR	20%	−0.0141	0.0545	0.0032	0.0511	—
K12	10%	0.50	MCAR	10%	−0.0076	0.0597	0.0025	0.0556	−3.99
K13	10%	0.50	MCAR	20%	−0.0178	0.0625	0.0082	0.0545	−13.18*
K14	10%	0.25	MAR	10%	−0.0030	0.0559	0.0108	0.0534	—
K15	10%	0.25	MAR	20%	−0.0076	0.0575	0.0253	0.0557	—
K16	10%	0.50	MAR	10%	−0.0036	0.0593	0.0132	0.0559	−21.09*
K17	10%	0.50	MAR	20%	−0.0069	0.0581	0.0274	0.0599	−43.86*
K18	20%	0.25	MCAR	10%	−0.0041	0.0552	0.0026	0.0526	−4.18
K19	20%	0.25	MCAR	20%	−0.0117	0.0591	0.0022	0.0528	−3.46
K20	20%	0.50	MCAR	10%	−0.0119	0.0624	0.0074	0.0588	−5.92*
K21	20%	0.50	MCAR	20%	−0.0197	0.0661	0.0121	0.0613	−9.72*
K22	20%	0.25	MAR	10%	−0.0026	0.0530	0.0110	0.0511	−17.58*
K23	20%	0.25	MAR	20%	−0.0044	0.0571	0.0241	0.0551	−38.52*
K24	20%	0.50	MAR	10%	−0.0097	0.0614	0.0168	0.0617	−13.40*
K25	20%	0.50	MAR	20%	−0.0118	0.0627	0.0289	0.0647	−23.14*

***Note.*** *IPD = Item Parameter Drift; Δb = drift magnitude in the b parameter; MCAR = Missing Completely at Random; MAR = Missing at Random; Bias_A and Bias_B = bias of the A and B equating constants; RMSE_A and RMSE_B = root mean square error of the A and B constants; RB_B = relative bias of the B constant (%).*

***Note.*** *RB_B values are reported only for conditions in which |B_true| ≥ 0.05; conditions falling below this threshold are marked (—) to avoid numerically unreliable results. Among the reported RB_B values, those exceeding the |RB_B| > 5% criterion* [55] *are flagged with an asterisk (), indicating meaningful bias.*

**Table 3:** Differences in bias and RMSE relative to the reference condition (K1) across 25 simulation conditions.

Condition	IPD Rate	Δb	Missing Mechanism	Missing Rate	ΔBias*A*	ΔRMSE*A*	ΔBias*B*	ΔRMSE*B*
**K1 (Reference)**	0%	0.00	—	0%	0.0000	0.0000	0.0000	0.0000
K2	10%	0.25	—	0%	−0.0004	0.0023	−0.0004	0.0003
K3	10%	0.50	—	0%	−0.0027	0.0055	0.0027	0.0032
K4	20%	0.25	—	0%	−0.0012	0.0041	0.0021	0.0037
K5	20%	0.50	—	0%	−0.0057	0.0121	0.0067	0.0087
K6	0%	0.00	MCAR	10%	−0.0079	0.0010	0.0034	−0.0003
K7	0%	0.00	MCAR	20%	−0.0138	0.0038	0.0046	−0.0002
K8	0%	0.00	MAR	10%	0.0004	0.0010	0.0101	0.0006
K9	0%	0.00	MAR	20%	−0.0058	0.0023	0.0258	0.0073
K10	10%	0.25	MCAR	10%	−0.0091	0.0034	0.0035	0.0005
K11	10%	0.25	MCAR	20%	−0.0154	0.0033	0.0039	0.0018
K12	10%	0.50	MCAR	10%	−0.0089	0.0085	0.0032	0.0063
K13*	10%	0.50	MCAR	20%	−0.0191	0.0112	0.0090	0.0052
K14	10%	0.25	MAR	10%	−0.0043	0.0047	0.0115	0.0041
K15	10%	0.25	MAR	20%	−0.0089	0.0063	0.0260	0.0064
K16*	10%	0.50	MAR	10%	−0.0050	0.0081	0.0139	0.0066
K17*	10%	0.50	MAR	20%	−0.0082	0.0069	0.0281	0.0106
K18	20%	0.25	MCAR	10%	−0.0054	0.0039	0.0033	0.0033
K19	20%	0.25	MCAR	20%	−0.0130	0.0079	0.0029	0.0035
K20*	20%	0.50	MCAR	10%	−0.0132	0.0112	0.0081	0.0095
K21*	20%	0.50	MCAR	20%	−0.0210	0.0148	0.0129	0.0120
K22*	20%	0.25	MAR	10%	−0.0039	0.0018	0.0117	0.0018
K23*	20%	0.25	MAR	20%	−0.0057	0.0059	0.0248	0.0058
K24*	20%	0.50	MAR	10%	−0.0110	0.0101	0.0175	0.0124
K25*	20%	0.50	MAR	20%	−0.0131	0.0115	0.0296	0.0154

***Note*** *. BiasA = bias of the A equating constant; BiasB = bias of the B equating constant; RMSE = root mean square error.*

***Note.*** *ΔBiasA = BiasA(condition) − BiasA(K1); ΔRMSEA = RMSEA(condition) − RMSEA(K1); ΔBiasB = BiasB(condition) − BiasB(K1); ΔRMSEB = RMSEB(condition) − RMSEB(K1). The K1 is the reference condition with all delta values equal to zero. Negative delta values reflect sampling variability in the reference condition and are not considered practically meaningful.*

***Note.*** * in the Condition column indicates that |RBB| > 5% [55]*, denoting meaningful bias in the B constant.*

### Effect of item parameter drift on bias and RMSE of equating constants

The first research question examines how the rate and magnitude of anchor-item parameter drift affect the accuracy of the A and B constants in the absence of missing data. For this purpose, conditions K1 (control), K2, K3, K4, and K5 were analyzed.

For the A constant (see Table 2), bias values ranged between −0.0043 and 0.0013, and no meaningful bias was observed in any condition. RMSE of constant A increased from 0.0512 in K1 to 0.0634 in K5; however, this increase remained limited. Relative bias values for A did not exceed the 5% threshold in any condition.

**Fig 1:**
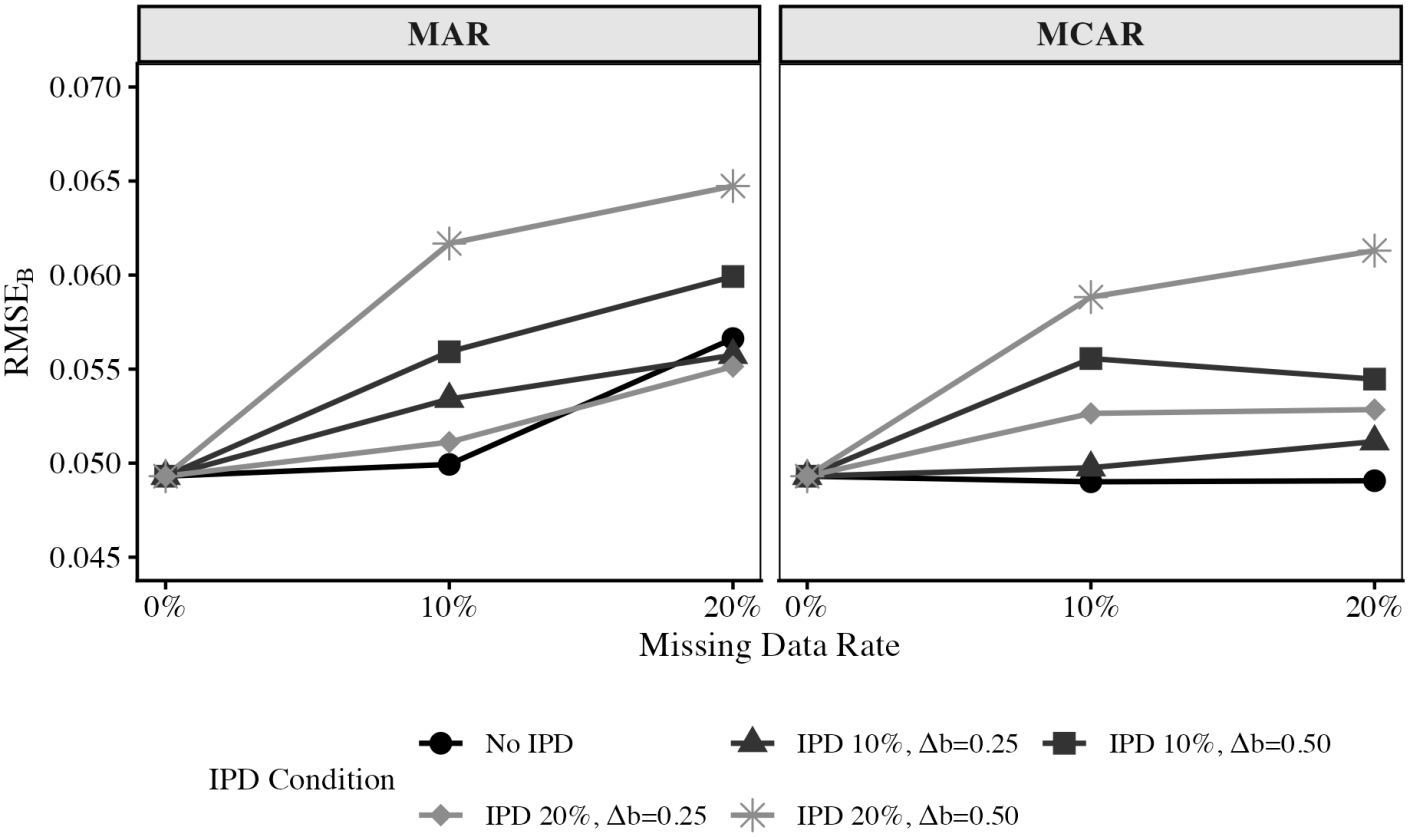
RMSE of the B Constant Across Missing Data Rates and IPD Conditions. RMSE values for the B equating constant under MCAR and MAR conditions across varying levels of missing data and IPD.

A similar pattern was observed for the B constant. Bias values were quite small (−0.0011 to 0.0060), and no statistically significant bias was detected. However, RMSE increased as the magnitude of IPD increased. The value, which was 0.0493 in K1, reached 0.0580 in K5 (IPD rate = 20%, Δb = 0.50). When the delta values in Table 3 are examined, ΔRMSE_B in K5 is 0.0087, corresponding to an increase of approximately 18% compared to K1. Relative bias (RB_B) could only be computed in conditions where the true value of B was meaningful; in those cases, values ranged from −2.25 to −4.78 and did not exceed the 5% threshold. This pattern is also visible in Fig 1. When the missing data rate is 0%, the starting points of all IPD conditions are very close, but the lines begin to diverge as the magnitude of drift increases.

Taken together, these findings indicate that item parameter drift alone, at least at the levels examined in this study, does not introduce substantial bias in the A and B constants, but it does increase estimation variability, particularly under moderate drift conditions (Δb = 0.50).

### Effect of missing data mechanism and rate on bias and RMSE of equating constants

The second research question focuses on the effects of the missing-data mechanism and rate in the absence of IPD. For this purpose, conditions K6, K7, K8, and K9 were compared with K1. The A constant remained largely stable in the presence of missing data. Bias values ranged from −0.0125 to 0.0018, and RMSE increased only slightly from 0.0512 in K1 to a maximum of 0.0550 in K7 (MCAR 20%) (see Table 2). No clear difference was observed between MCAR and MAR conditions in terms of the A constant.

For the B constant, however, the two mechanisms show a clear divergence. Under MCAR conditions (K6–K7), bias values (0.0027 and 0.0039) remained very close to that of K1 (−0.0007), and RMSE values stayed within a narrow range (0.0490–0.0491). Under MAR conditions, the pattern changes; bias increased to 0.0094 in K8 (MAR 10%) and to 0.0251 in K9 (MAR 20%). The delta values in Table 3 further highlight this difference, with ΔBias = 0.0258 and ΔRMSE = 0.0073 in K9. Since there is no true location difference between forms in conditions without IPD, the true population value of B is zero; therefore, relative bias could not be computed due to division by zero, and interpretations were based on absolute Bias_B values. Fig 1 also visually supports this distinction. In the MCAR panel, the line for the no-IPD condition remains nearly flat as the missing rate increases, whereas in the MAR panel, a clear upward trend is observed.

**Fig 2:**
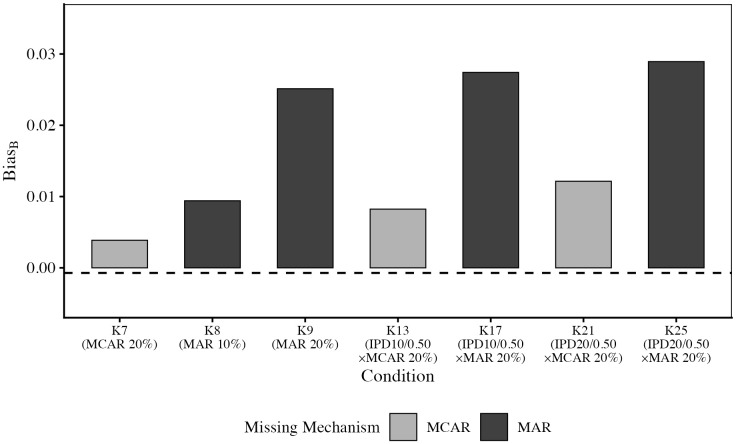
Bias in the B Constant Across Selected MCAR and MAR Conditions. Bias values of the B equating constant under selected missing data and IPD conditions across MCAR and MAR mechanisms.

A similar pattern can be seen in Fig 2, where K7 (MCAR 20%) remains close to the reference line, while K8 and K9 (MAR) show a noticeable increase.

These findings suggest that the MCAR mechanism does not systematically affect the B constant, whereas the MAR mechanism, especially at a 20% missing rate, introduces a notable positive bias. This pattern likely reflects the systematic loss of lower-ability individuals under MAR conditions.

### Combined effects of IPD and missing data

The third research question examines whether the combined effect of IPD and missing data exceeds the effect of each factor alone. For this purpose, conditions K10–K25 were analyzed.

For the A constant, combined conditions led to only a limited increase in RMSE, mainly when the missing rate was higher. The highest RMSE value (0.0661) was observed in K21 (IPD 20%, Δb = 0.50 × MCAR 20%), corresponding to a ΔRMSE of 0.0148 relative to K1. Bias did not show any meaningful variation across combined conditions.

For the B constant, the pattern is more pronounced. Under MCAR conditions (K10–K13, K18–K21), bias remained relatively low; however, RMSE increased as both IPD magnitude and missing rate increased. In K21, RMSE was 0.0613, with a ΔRMSE_B of 0.0120. According to the relative bias values of B, K20, and K21, which exceeded the threshold for meaningful bias (see Table 2; −5.92* and −9.72*, respectively).

Under MAR conditions (K14–K17, K22–K25), the pattern becomes more striking. Bias of the B constant reached 0.0274 in K17 and 0.0289 in K25. These conditions also produced the highest RMSE values; in K25, RMSE was 0.0647, with a ΔRMSE of 0.0154. Most of the conditions marked with an asterisk in Table 3 involve the combination of moderate missing rates and moderate IPD, suggesting that the joint effect of these factors is not merely additive but reflects an interaction. This is also evident in Fig 1. When the two panels are compared, the distance between lines is clearly larger under MAR than under MCAR conditions. Fig 2 shows a similar pattern, with MAR conditions (K17, K25) displaying notably higher bias than MCAR conditions (K13, K21).

### Role of multiple imputation in reducing equating error

The fourth research question investigates the extent to which multiple imputation reduces equating error under missing data and IPD conditions. A direct comparison with complete data conditions (i.e., without MICE) was not possible, since MICE was applied in all conditions with missing data. Therefore, its effectiveness can only be evaluated indirectly by comparing MCAR and MAR conditions.

Under MCAR conditions, the bias values of the B constant remain close to zero across all missing rates (K6: 0.0027; K7: 0.0039; see Table 2), suggesting that MICE effectively reduced equating bias when missingness was completely random. In contrast, under MAR conditions, bias increased noticeably with missing rate (K8: 0.0094; K9: 0.0251; see Table 2), indicating that MICE does not fully eliminate bias arising from systematic missingness.

## Discussion and conclusion

In this study, the effects of IPD, missing data mechanisms (MCAR, MAR), and missing data rates (10% and 20%) on the accuracy of the SL transformation constants (A and B) in IRT-based test equating were examined through simulation. When the findings were considered in light of the four research questions, it became clear that the A constant remained largely stable across all conditions. The main pattern, however, emerged in the B constant, and was primarily driven by the missing-data mechanism.

With respect to the first research question, no meaningful bias was detected in either the A or B constants under conditions that included only IPD (K1–K5). Although RMSE values increased slightly as the magnitude of IPD increased, this increase did not reach a level of practical concern. This result partially aligns with the patterns reported by Wells et al. [14] and Han et al. [30]. Those studies similarly indicated that IPD alone does not substantially distort equating constants, although estimation errors tend to accumulate as the magnitude and proportion of drift increase. In the present study, the relatively moderate levels of IPD (10% and 20%) and drift magnitudes (0.2–0.5) suggest that deterioration in equating accuracy is largely a function of drift size, with stronger effects likely to emerge at higher levels [32].

Regarding the second research question, MCAR and MAR mechanisms produced clearly different effects on the B constant. Under MCAR conditions, bias values for B remained close to zero. In contrast, under MAR conditions, particularly at the 20% missing rate, a noticeable positive bias was observed. This finding indicates that when missingness is completely random, parameter estimation is largely preserved. However, when missingness is systematically related to ability, the B constant becomes measurably distorted.

Missing data mechanisms are among the key factors influencing equating accuracy. MAR is more complex than MCAR because the probability of missingness depends on the observed variables. When appropriate methods are not employed, this complexity can lead to higher levels of error and bias in parameter estimation. Indeed, the missing-data literature emphasizes that, under MAR conditions, violations of model assumptions or the use of inadequate methods can increase estimation error [19,56]. In this respect, correctly identifying the missing-data mechanism and handling it with appropriate techniques is critical for accurate equating results. The findings of the present study confirm this pattern within the context of the 3PL model and the SL method. Under MAR conditions, the dependence of missingness on individuals’ ability levels can bias both item and ability parameter estimates, ultimately reducing equating accuracy [21].

For the third research question, the combined effect of IPD and missing data produced greater bias than either factor alone. This interaction was especially pronounced in combinations involving MAR and moderate levels of IPD, whereas it remained much more limited in conditions that included MCAR. The highest levels of error were observed in condition K25 (IPD 20%, b = 0.50 × MAR 20%), where the bias for the B constant reached 0.029 and the RMSE value was 0.065. This pattern suggests that uncertainty stemming from two sources may reinforce each other, particularly when missingness is concentrated among lower-performing examinee groups, thereby amplifying the effect of IPD. To the best of our knowledge, no study in the literature directly examines the joint effect of these two factors. Therefore, the present findings underscore the importance of considering sources threatening equating accuracy in combination rather than in isolation.

Regarding the fourth research question, the effectiveness of MI can be indirectly evaluated by comparing MCAR and MAR conditions. Under MCAR, MI largely compensated for the B constant. Under MAR, however, bias continued to increase to a notable extent, especially when moderate levels of missingness and IPD co-occurred. MI, based on Rubin’s [57] rules, is known to improve parameter estimation under ignorable missing-data conditions, yet it does not fully eliminate the effects of systematic missingness patterns. While MCAR does not systematically distort parameter estimates, MAR conditions are known to produce noticeable bias. The present study shows that this general pattern also holds in the context of equating and highlights cases in which MAR-related bias cannot be fully corrected through MICE. These findings suggest that MICE was more effective under MCAR conditions than under MAR conditions, particularly when moderate levels of missingness (20%) and IPD (Δb = 0.50) co-occurred.

Overall, the findings of this study point to three main conclusions. First, the missing-data mechanism, particularly MAR, can introduce systematic bias into equating constants. Second, estimation error increases markedly as the rate of missing data rises. Third, although the effect of IPD alone is limited, it becomes considerably stronger when combined with MAR.

These findings also have several practical implications for operational test equating. First, missing-data patterns in operational assessments should be systematically monitored and documented prior to equating, as the mechanism, not only the rate, determines the extent of equating error. Therefore, in large-scale assessments, missing-data patterns should be systematically monitored and reported. Second, although MICE reduces error under MAR conditions, it does not eliminate it entirely. In the present study, imputation was conducted using item responses only, without incorporating person-level covariates. Drawing on general MI theory, including performance-related auxiliary variables, such as total score, in the imputation model may help reduce bias under MAR conditions by better capturing the cause of missingness [58]; however, the extent to which this approach improves equating accuracy warrants empirical investigation in future studies. Third, in the presence of IPD, the quality control of the anchor item set is critically important. Identifying and removing drifting anchor items prior to equating appears essential for limiting equating error under both MCAR and MAR conditions.

Several limitations of the present study should be acknowledged. First, the MAR mechanism was operationalized by assigning differential missingness probabilities based on examinees’ latent ability (θ). Although this approach is commonly used in IRT simulation studies and reflects realistic omission behavior in testing contexts [15,21], the resulting condition is more appropriately interpreted as a near-MAR mechanism rather than a strictly observed-variable MAR process. Future studies may extend this framework by incorporating directly observed auxiliary variables or by examining fully MNAR conditions.

A second limitation concerns the imputation strategy. In the present study, MICE was implemented using item response data only, without including person-level auxiliary variables such as total score or subgroup membership. Including such variables may improve the plausibility of the MAR assumption and further reduce residual bias under missing data conditions [56,58]. Future research may also consider comparing MICE with no-imputation or complete-case approaches to evaluate the relative effectiveness of different missing data handling strategies in IRT equating contexts.

Additional design assumptions should also be considered when interpreting the generalizability of the findings. First, the guessing parameter (c) was fixed at 0.20 across all items, which simplified estimation but does not reflect the item-level variability in pseudo-guessing typically observed in operational testing programs. Second, item parameter drift was simulated exclusively in the positive direction to facilitate the detectability of equating error and isolate the effect of drift magnitude. Accordingly, the findings may be most applicable to testing conditions in which drift occurs predominantly in a single direction. Future studies should examine how item-level variation in c parameters and bidirectional drift patterns interact with missing data mechanisms to influence equating accuracy.

The present study examined the joint effects of item parameter drift and missing data on IRT-based test equating accuracy within a unified simulation framework. Three main conclusions emerge from the findings. First, the missing data mechanism, rather than the rate of missingness alone, is the primary driver of equating error, with MAR conditions introducing substantial positive bias in the B constant that cannot be fully mitigated by MICE. Second, item parameter drift alone produced relatively limited bias at the levels examined, but amplified equating error considerably when combined with MAR. Third, while MICE effectively reduces error under MCAR, its performance is limited under systematic missingness conditions. Together, these findings underscore the importance of diagnosing missing data mechanisms prior to equating, monitoring anchor item stability, and developing more robust imputation strategies for operational testing programs, particularly in psychological assessment contexts where equating errors may affect individual-level decisions.
